# Influence of Fine Management Combined With PDCA Cycle Method on Disinfection Qualified Rate and Performance Grade of Ophthalmic Precision Instruments

**DOI:** 10.3389/fsurg.2022.856312

**Published:** 2022-03-15

**Authors:** Fanli Zeng, Xiuling Wang, Yan Gao, Ling Hu

**Affiliations:** ^1^Department of Anesthesiology, Chongqing General Hospital, Chongqing, China; ^2^Department of Ophthalmology, Chongqing Banan District People's Hospital, Chongqing, China; ^3^Central Sterile Supply Department, Chongqing General Hospital, Chongqing, China; ^4^Central Sterile Supply Department, Chongqing Hospital of Traditional Chinese Medicine, Chongqing, China

**Keywords:** ophthalmic precision instruments, fine management, PDCA cycle method, disinfection qualified rate, performance grade, failure mode and effect analysis

## Abstract

**Objective:**

The aim of this study is to explore the influence of fine management combined with the plan-do-check-action (PDCA) cycle method on the management of ophthalmic precision instruments.

**Methods:**

The ophthalmic precision instruments centralized in the disinfection supply room of our hospital were selected as the research objects and divided into groups A and B. Traditional instrument management method was adopted in group A, and fine management combined with the PDCA cycle method based on the group A was adopted in group B. The instrument management risk scores, the qualified rate of disinfection, instrument performance grade, and incidence of toxic anterior segment syndrome (TASS) of the two groups were compared.

**Results:**

The risk scores of instrument management and incidence of TASS in group B were lower than those in group A (*p* < 0.05). The qualified rate of disinfection and instrument performance grades in group B were higher than those in group A (*p* < 0.05).

**Conclusion:**

Fine management combined with the PDCA cycle method can improve the qualified rate of disinfection of ophthalmic precision instruments, optimize the performance of instruments, reduce the risk of instrument management, and reduce the incidence of TASS.

## Introduction

Disinfection supply room is a special department of hospital, and its work quality can directly affect nursing work and medical safety, which is closely related to nosocomial infection rate, and makes the clinical nursing management of disinfection supply room have higher requirements. With the continuous development of medical technology, the skill of ophthalmic surgery is gradually improved, and fine ophthalmic instruments are the prerequisite for the successful completion of ophthalmic surgery. However, ophthalmic precision instruments have a complicated structure, fast turnover, high price, high precision, and high maintenance requirements which are difficult to clean and require high disinfection and maintenance (1, 2). If the ophthalmic precision instruments are not handled in time and maintained properly in the cleaning and disinfection process, it is easy to cause the corrosion and damage of the instruments and destroy the performance, which will not only affect the operator's operation process, but also possibly lead to the phenomenon of intraocular infection, which will adversely affect the surgical effect (3, 4). The research shows that infections caused by incomplete sterilization of surgical instruments account for about 20% of patients with infections after ophthalmic surgery (5). Therefore, attention should be paid to the treatment of ophthalmic precision instruments in clinic. Strengthening the cleaning and disinfection of ophthalmic instruments plays an important role in improving the safety of surgery.

At present, people's requirements for medical services are gradually increasing, and nursing work is gradually becoming more refined. Fine management is an intervention means of fine care for every detail, which can carry out quality management from many angles, such as sorting, rectification, treatment, and supervision, focusing on finding problems in detail, actively looking for reasons, formulating a series of management procedures, and comprehensively supervising the operation of every link, so as to achieve the purpose of solving problems (6). Fine management through process control and continuous improvement is helpful to improve the management efficiency of hospitals, further coordinate and unify the management mechanism, and provide better service intervention for patients, thus effectively avoiding nursing defects and improving the clinical work quality (7). Plan-do-check-action (PDCA) cycle method is a working procedure of all-round quality management, which divides quality management into four stages: planning, implementation, inspection, and treatment (8). In the PDCA cycle, each stage promotes each other, interlocks with each other, and spirals upward. The purpose of implementation is to reduce the shortage of management schemes and improve the overall work quality through a continuous cyclic operation (9).

At present, how to use effective methods to avoid the risk factors of eye infection and improve the qualified rate of disinfection of instruments has become a hot issue for the staff of disinfection supply room and medical staff. Therefore, to explore whether the fine management combined with the PDCA cycle method is practical in the management of ophthalmic precision instruments, we have carried out the following research.

## Materials and Methods

### Object

In this study, 80 sets of ophthalmic instrument packages centralized from the disinfection supply rooms of two hospitals were selected as the research objects. A number of 40 sets of ophthalmic instrument packages (785 ophthalmic precision instruments) randomly selected from July 2020 to December 2020 were taken as group A, and 40 sets of ophthalmic instrument packages (791 ophthalmic precision instruments) randomly selected from January 2021 to June 2021 were taken as group B. Inclusion criteria were as follows class I ophthalmic precision instruments; the use time of surgical instruments was <2 years; the information of surgical instruments was complete. Exclusion criteria were experienced derusting treatment, the fact that before the study, it was defective or the performance was greatly reduced, and surgical instruments did not meet the management requirements of the disinfection supply room.

### Methods

#### Group A

A traditional instrument management method was adopted. Cleaning and disinfection of instruments adopted traditional manual cleaning, assigned personnel to recycle the used instruments after the operation, and classify the instruments before cleaning. According to the characteristics of ophthalmic precision instruments, prewashing, soaking, rinsing, drying, lubrication, disinfection, and sterilization were carried out in turn. The processed instruments were inspected regularly, the existing problems were found in time, and corresponding measures to intervene were formulated.

#### Group B

On the basis of group A, fine management combined with PDCA cycle method was adopted.

(1) ① Determining the goals that need to be achieved. It was agreed that the qualified rate of instruments cleaning quality should reach above 90%; hence, the two hospitals adopted the same model and established a fine management joint PDCA cycle method team, which included ophthalmology specialist nurses, and disinfection supply center nurses. The cleaning station was moved to the ophthalmology department, and cleaning and sterilization were performed at the disinfection supply center. After the group was established, specialized knowledge training and technical operation assessment were actively carried out, so as to inspect the group members' mastery of professional skills. The problems existing in the management of ophthalmic precision instruments were identified, such as the lack of continuous and effective monitoring of the cleaning quality of instruments, the lack of refinement in the process of different types of instruments, and the failure to strictly follow the cleaning process, when the workload was heavy. The members of the group were informed of relevant knowledge and matter needing attention in disinfection management of ophthalmic precision instruments, and the rules and regulations of hospital disinfection supply room were further improved. The nurses in the disinfection and supply room completed the classification of instruments, clarified them into books, and placed them in the recycling and packaging area. ② The reasons for unqualified cleaning and disinfection quality of instruments in the past were clarified, and the main causes were analyzed and summarized. ③ A fine management system of ophthalmic precision instruments was established.
(2) ① A group meeting was held, division of labor was formulated, responsibilities were clarified, and the awareness of infection prevention and control of among medical staff was improved. ② Then, through the combination of the actual instrument and the picture of the instrument, the department staff was trained on the related operation links of instrument cleaning and disinfection, the atlas of ophthalmic precision instruments was made, name and performance of the instruments were indicated, and the nursing staff was asked to master the cleaning points, such as matters needing attention in packaging and maintenance, disinfection and sterilization methods of ophthalmic precision instruments, etc. ③ When checking the instruments, nurses should be paid attention to whether the instruments were in good condition, whether there was any defect, whether the alignment was tight, and whether the functions were good. The instrument was handled with care to prevent its precision from being damaged. A special treatment table for ophthalmic instruments was set up and they were treated separately from ordinary instruments. ④ According to the characteristics of the instruments, the cleaning process was established. The scissors, pliers, tweezers, and cavity instruments were cleaned manually, when washing the instruments manually, the action should be gentle, and cleaning tools, such as regular brushes and sponge brushes, should be used correctly. The instruments with difficult cleaning and complicated structure were cleaned ultrasonically, during which they were first pre-cleaned by hand after which the instrument was disassembled. The shaft joint was then opened and then put it into an ultrasonic cleaning machine. After cavitation and vibration for 2–3 min, the joints were cleaned by the hand-cleaning method, and the cavity was rinsed with a high-pressure water gun. When cleaning cavity instruments, appropriate cleaning agents should be chosen according to the instructions of device manufacturers to remove residual substances on the wall of the tube and then washed with pure water. Instruments that were not resistant to moist heat disinfection should be soaked in 75% ethanol for disinfection and dried by an air gun. ⑤ The cleaning methods of ophthalmic precision instruments with high unqualified rates were analyzed. The eyelid opener should focus on brushing the eyelid opening to remove mucus and glue. Small scissors should focus on cleaning the blade and the adhesive should be wiped off with alcohol gauze. Scleral presser should focus on brushing the top pressure and removing mucus. During microshear cleaning, the joints should be completely opened. The ultrasonic emulsification tube and IA head should be cleaned with a water gun and an air gun. When cleaning the effusion box, the cavity should be filled with water, water should be poured while shaking, rinsing, and disinfecting. ⑥ When cleaning, the operator should move gently without violence, and used soft tools to clean the occlusal parts or parts with sharp tips. A special fine basket with a built-in silicone pad to hold ophthalmic precision instruments should be used, and the tip of the instrument should face upward to prevent the tip from being damaged. When packaging instruments, fine-grained baskets, precision instrument boxes, and protective sleeves that match the instruments should be used to avoid instruments' damage caused by the instrument bag being squeezed or inverted. ⑦ After cleaning and disinfection of ophthalmic precision instruments, the cleaner shall sign and improve the handover register. When packing, silica gel head and plastic protective sleeve should be used, and when transporting, a special container to prevent the instrument from being damaged should be used.
(3) Check the designed daily monitoring record of cleaning and disinfection quality of ophthalmic precision instruments, appoint team members to check the cleaning and disinfection quality of the instruments every day, and check whether the cleaning effect of the surfaces, shaft joints, tooth slots, and other parts of the instruments was up to standard, if the surface of the surgical instrument was smooth and there was no residual bloodstain or rust stain, it was qualified, and the registration was unqualified. The sterilized instruments should be checked one by one for sharpness, good function, defects, or serious corrosion. If the instruments were defective or improperly maintained, the nursing staff should report for repair or update the instruments in time.
(4) A group meeting should be held once a month, the problems existing in the inspection should be summarized, analyzed and the reasons for unqualified disinfection quality of instruments should be found. Specific and effective measures to solve the problems should be put forward and the corresponding work contents should be adjusted. A refined work plan should be formulated for all employees to prevent the same work defects from recurring, or the unresolved problems will roll into the next PDCA cycle.

### Observation Index

(1) The basic information of ophthalmic precision instruments, such as service time of instruments and instrument type of the two groups, were recorded.
(2) The management risks of ophthalmic precision instruments according to the failure mode and effect analysis (FMEA) evaluation standard, including unqualified cleaning quality, instrument defect, improper handover of the instrument, and difficulty in instrument turnover, were evaluated. The evaluation method was divided into severity (S), occurrence (O), and detection ability (D). On calculating the risk priority number (RPN) = S × O × D, with a total score of 1–1,000 points, we defined RPN ≥ 150 points as high risk. The higher the score, the higher the risk of management of ophthalmic precision instruments. The specific scoring content is shown in Table 1.
(3) Different methods were used to evaluate the qualified rate of disinfection of ophthalmic precision instruments. ① Magnifying glass detection: The smoothness of the surface of the instrument was observed under a 5x magnification lens, and whether there was any contamination residue. The external surface was clean and free of pollution residue, which was qualified; ② Microbial culture detection: sterile cotton swabs were sampled on the instruments for bacterial culture, and the number of bacterial flora was calculated. The number of bacteria in a single instrument was <20 cfu, which was qualified; ③ Adenosine triphosphate (ATP) bioluminescence detection: the irrigating apparatus was purified five times, irrigating fluid was collected, the irrigating fluid with 3M ATP fluorescence detection swab was dipped, and the ATP fluorescence value was measured. Relative light unit ≤ 150 was qualified; ④ Jerry test paper method detection: dip the washing solution with Jerry test paper and observe the color change of the test paper. After 1 min, the yellow test paper was qualified.
(4) The performance questionnaire of ophthalmic precision instruments made by our hospital was used for evaluation. The score range was 4–12 points, 4 points: four levels, 5–6 points: three levels, 7–9 points: two levels, and 10–12 points: one level. The higher the score, the better the instrument performance. Specific scoring content is shown in Table 2.
(5) A total number of 200 patients with ophthalmic surgery were selected from each of the two groups, and the incidence of toxic anterior segment syndrome (TASS) during the management of the two groups was recorded. TASS diagnostic criteria were as follows: ① 22–24 h after cataract surgery; ② decreased vision, but no obvious pain, or mild pain; ③ the patient had diffused corneal edema accompanied by ciliary hyperemia, and the endothelial cell loss rate was >70%; ④ a large amount of cellulose protrudes into the anterior atrial abscess, and the pupil was irregularly dilated; ⑤ there was an inflammatory reaction in the anterior segment, but no obvious inflammatory reaction in the posterior segment. ⑥ Gram staining and bacterial culture were performed on aqueous humor and vitreous, and both the results were negative.

**Table 1:** FMEA evaluation standard.

**S evaluation**	
1 point	Without any effect on the instrument.
2–3 points	Affect the instruments, not affect the normal use.
4–6 points	The instrument is inconvenient to use, and the doctor is slightly dissatisfied.
7–8 points	Doctors are seriously dissatisfied with the status quo of instruments.
9–10 points	Doctors are seriously dissatisfied, and the status quo of instruments may cause medical accidents.
**O evaluation**	
1 point	Almost impossible to happen.
2 points	It may happen slightly.
3 points	Could happen.
4–6 points	Occasional, but unlikely.
7–8 points	Be of frequent occurrence.
9–10 points	Almost inevitable.
**D evaluation**	
1–2 points	Almost certainly.
3–5 points	Good detection means exist.
6–8 points	May be detected.
9 points	It is very likely that it will not be detected.
10 points	Can't be detected with high probability.

**Table 2:** Ophthalmic precision instrument performance questionnaire.

**Scissors type**	
**1 point**	**Cut thin cotton sheets, fail to cut them neatly and/or are sticky**.
**2 points**	Cut neatly without sticking, dull.
3 points	Cut it neatly without sticking.
**Clamp type**	
1 point	The palm of your hand is slapped or thrown from the air, and it automatically pops open and/or cannot be completely closed.
2 points	It doesn't bounce off automatically, it can clamp the No. 1 thread end, and there is a sense of pause when used.
3 points	It does not bounce off automatically. When it is completely closed, it clamps the No. 1 thread end and does not fall off.
**Tweezers type**	
1 point	Touched by hand when closed, rough, staggered and/or defective.
2 points	Smooth, staggered or defective.
3 points	Smooth, without misalignment and defects.
**Cavity type**	
1 point	Visually, there is dirt, blood, and rust. Under the condition that the cavity is not dried, the water in the cavity is blown to a clean white gauze with an air gun, and the color of the gauze changes obviously.
2 points	Visually, there is no dirt, blood, and rust, but the color of gauze changed slightly.
3 points	Visually, there is no dirt, blood, and rust, and the gauze is clean as before, with the same color.

### Statistical Methods

The SPSS 22.0 software was used, the measured data were expressed by $\bar{x}$ ± s, and the *t*-test was used for comparison. The counting data were expressed as %, and the χ^2^ test was used for comparison; *p* < 0.05, the difference was significant.

## Results

### Basic Information of Ophthalmic Precision Instruments

There was no significant difference in the basic information of ophthalmic precision instruments between the two groups (*p* > 0.05), as shown in Table 3.

**Table 3:** Basic information of ophthalmic precision instruments (*n*, $\bar{x}$ ± s, %).

**Group**	**Service time of instruments (months)**	**Instrument type**				
		**Scissors type**	**Clamp type**	**Tweezers type**	**Cavity type**	**Other type**
Group A (*n* = 785)	13.18 ± 2.05	121 (15.41%)	164 (20.89%)	175 (22.29%)	149 (18.98%)	176 (22.42%)
Group B (*n* = 791)	13.26 ± 1.97	130 (16.43%)	152 (19.22%)	177 (22.38%)	153 (19.34%)	179 (22.63%)
*χ^2^ / t* value	0.789	0.845				
*P*-value	0.429	0.932				

### Comparison of Instrument Management Risk Scores Between Two Groups

**Figure 1:**
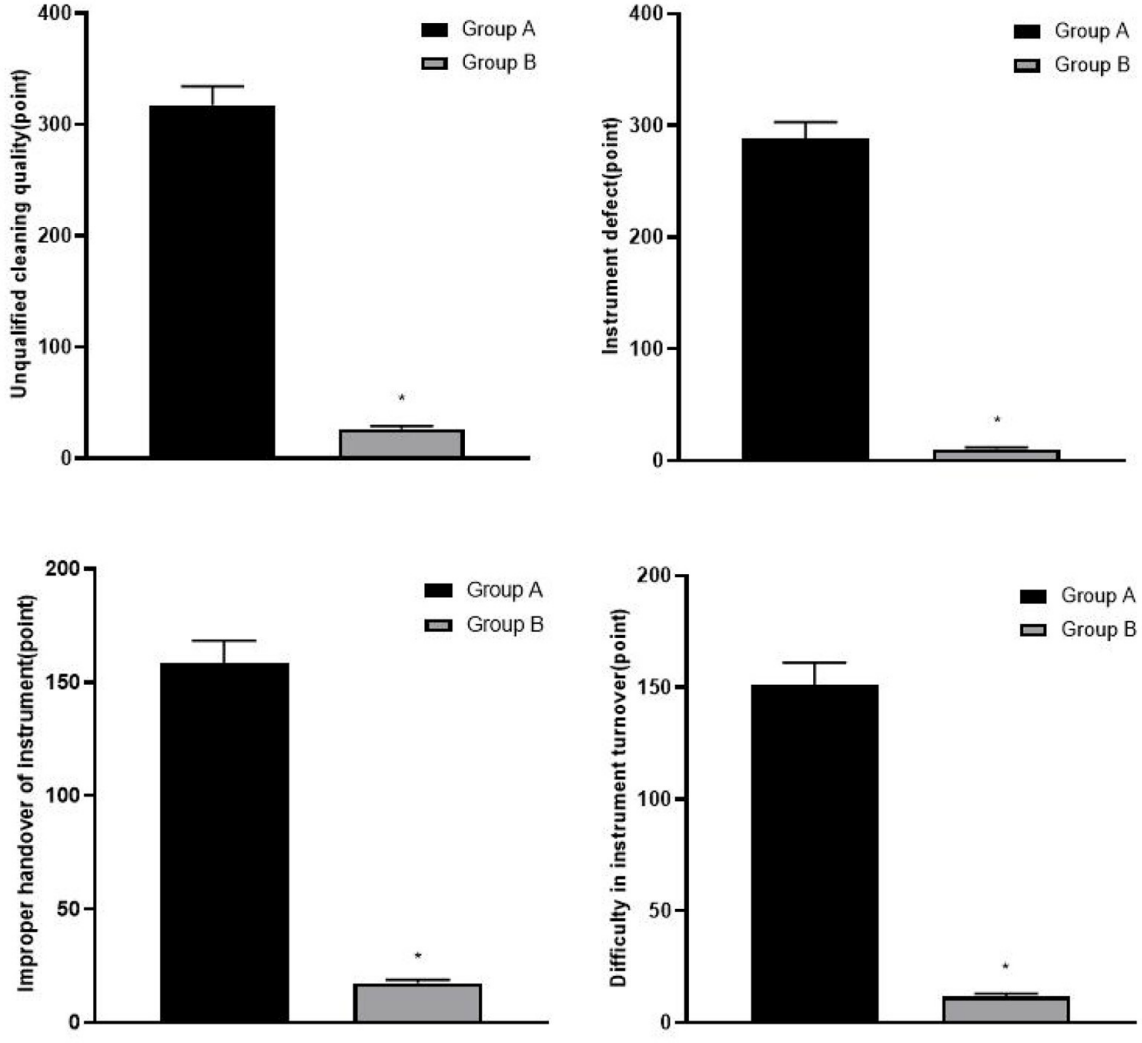
Comparison of risk scores of instrument management between two groups, compared with group A, *p < 0.05.

The risk scores of instrument management in group B were lower than those in group A (*p* < 0.05), as shown in Figure 1.

### Comparison of Qualified Rate of Disinfection Between Two Groups

The qualified rate of disinfection in group B was higher than that in group A (*P* < 0.05), as shown in Table 4.

**Table 4:** Comparison of qualified rate of disinfection between two groups (*n*, %).

**Group**	**Magnifying glass detection**	**Microbial culture detection**	**ATP bioluminescence detection**	**Jerry test paper method detection**
Group A (*n* = 785)	776 (98.85%)	773 (98.47%)	743 (94.65%)	712 (90.70%)
Group B (*n* = 791)	791 (100.00%)	790 (99.87%)	768 (97.09%)	742 (93.81%)
*χ^2^* value	9.121	9.470	5.945	5.318
*P*-value	0.003	0.002	0.015	0.021

### Comparison of Instrument Performance Grade Between Two Groups

**Figure 2:**
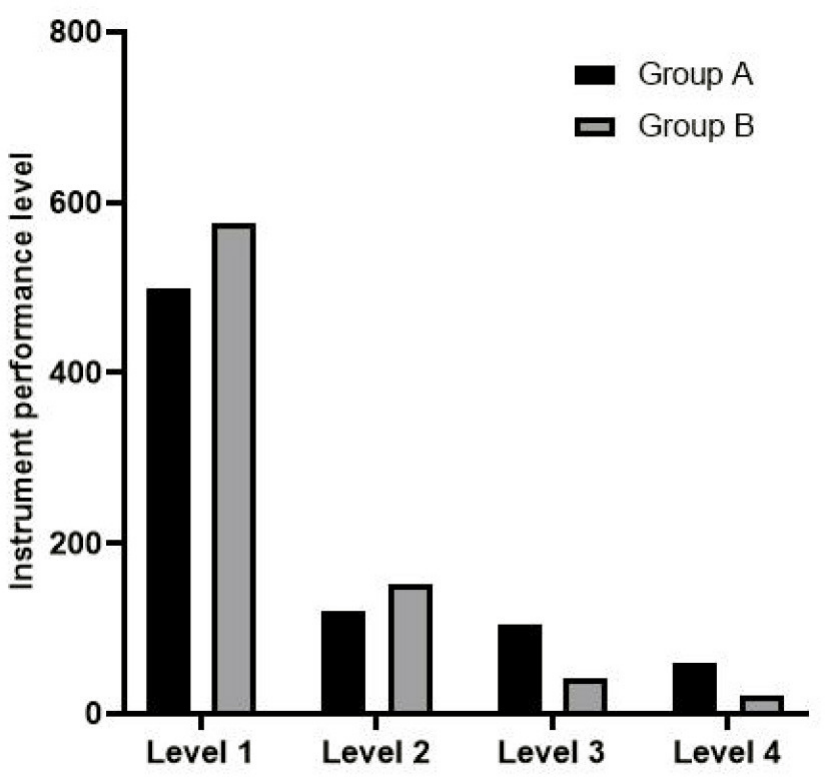
Comparison of instrument performance grade between two groups.

The instrument performance grade in group B was better than that in group A (rank sum test *z* = 4.012, *p* < 0.05), as shown in Figure 2.

### Comparison of the Incidence of TASS Between Two Groups

The incidence of TASS in group B (0.50%) was lower than that in group A (3.50%) (*p* < 0.05), as shown in Table 5.

**Table 5:** Comparison of the incidence of TASS between two groups (*n*, %).

**Group**	**Number of TASS cases**	**Incidence of TASS**
Group A (*n* = 200)	7	3.50%
Group B (*n* = 200)	1	0.50%
*χ^2^* value		4.592
*P*-value		0.032

## Discussion

People's eye structure is complex and there are many kinds of diseases. To meet the needs of various ophthalmic operations, ophthalmic surgical instruments are developing toward complexity and refinement. This not only brings convenience to ophthalmic surgery, but also increases the management difficulty of ophthalmic precision instruments. Ophthalmic precision instruments are easily damaged, and have high maintenance requirements. If the bacteria of the instruments are not completely eliminated or the cleaning quality is not up to standard, it will adversely affect the operation effect (10, 11). Therefore, it is of great significance to find a scientific instrument management method to promote the smooth development of ophthalmic surgery.

Although the traditional instrument management method adopted in the disinfection supply room can achieve a good management effect, there are relatively many problems and high instrument loss. Improper management can damage ophthalmic precision instruments, cause waste of medical resources, may also damage patients, affect the safety of clinical use, and cause medical accidents and medical disputes (12). Fine management is a management means to formulate a series of detailed operations according to the actual situation, which can identify, observe, extend the details, and improve the overall medical quality (13). This model starts with details, with the idea of improving management level, and continuously optimizes the intervention scheme, which can meet the requirements of managers to the greatest extent, and is professional and comprehensive (14). The PDCA cycle method includes small cycle and large cycle, which is a process of dynamic cycle rising step by step, and is managed repeatedly (15). Before the implementation of PDCA cycle method, it is necessary to set up an activity group to find and analyze the causes of past management errors, and then determine the expected goals, formulate improvement plans, and then carry out implementation and inspection. Finally, evaluate the effectiveness of improvement measures, think about unresolved problems, and enter the next round of PDCA cycle (16). Fine management and PDCA cycle method have received extensive attention in clinic. Therefore, we applied the two methods to the management of ophthalmic precision instruments and achieved good results.

Toxic anterior segment syndrome is an acute noninfectious postoperative inflammatory reaction, and it has become one of the common complications after eye surgery. The main clinical manifestations are diffused corneal edema and irregular pupil enlargement caused by anterior chamber cellulose exudation. At present, it is generally believed that the occurrence of TASS is closely related to the imperfect cleaning technology and incomplete disinfection of surgical instruments. In this study, compared with group A, group B has a lower risk score of instrument management, lower incidence of TASS, and better-qualified rate of disinfection and instrument performance. This suggests that fine management combined with PDCA cycle method can improve the qualified rate of disinfection of ophthalmic precision instruments, optimize the performance of instruments, reduce the risk of instrument management, and reduce the incidence of TASS. Fine management can optimize the operation process of each link, establish and improve the instrument maintenance measures, control the occurrence of risk factors, effectively improve the instrument performance, reduce the instrument loss, significantly improve the cleaning quality and functional quality of instruments, and ensure smooth operation. The quality of medical staff is an important prerequisite for fine management. Fine management can actively carry out specialized knowledge training and inform team members of relevant knowledge and precautions in disinfection management of ophthalmic precision instruments, so as to improve the awareness of prevention and control of infection among medical staff and ensure the cleaning quality and performance of ophthalmic surgical instruments in good condition, which is very important to ensure the safety of surgery, prolong the service life of instruments, and reduce medical costs. In the fine management, by optimizing the process of recycling, cleaning, packaging, sterilization and transportation of instruments, and strengthening the disinfection management of the storage environment of instruments, we can not only avoid the errors of instruments, but also avoid the pollution and damage of instruments. Cleaning instruments by combining both manual and utrasonic methods is time- and labor-saving, quick and convenient, and can thoroughly clean small stains. The administrator needs to carefully check whether each instrument is in good condition and without defects, strengthen the daily maintenance of precision instruments, and ensure that the instruments are in the best use state (17–19). The PDCA cycle method adopts different cleaning methods for different instrument fineness. Solid instruments are cleaned by hand, instruments with complicated structures are disassembled and cleaned ultrasonically, and cavities are repeatedly washed by a high-pressure water gun. When cleaning, appropriate cleaning agents are injected into the cavity. Additionally, nurses focus on cleaning and disinfection of instruments with high unqualified rate, thus effectively avoiding potential safety hazards in the management process and providing effective guarantee for improving the quality of instruments. In the process of implementing the PDCA cycle method, after each cycle, the group will integrate the problems existing in the instrument management, analyze and find out the reasons, formulate targeted improvement plans, adjust the work specifications, supervise and inspect, evaluate the improvement measures after inspection, and summarize the management experience. In addition, the manager will modify the measures with inaccurate management effect and incorporate new problems into the next PDCA cycle, so as to improve the instrument management quality in the continuous cycle and achieve the purpose of functional quality control (20–22). According to the uniqueness of ophthalmic precision instruments, fine management, and the PDCA cycle method are carried out on the basis of routine management, and the quality of ophthalmic precision instrument management has been greatly improved.

## Conclusion

To sum up, fine management combined with the PDCA cycle method can improve the qualified rate of disinfection of ophthalmic precision instruments, optimize the performance of instruments, reduce the risk of instrument management, and reduce the incidence of TASS.

## Data Availability Statement

The original contributions presented in the study are included in the article/supplementary material, further inquiries can be directed to the corresponding author.

## Author Contributions

The manuscript was written by FZ and LH was the supervisor of the whole study. All authors of this study have contributed equally and have done work of equal value to this study.

## Conflict of Interest

The authors declare that the research was conducted in the absence of any commercial or financial relationships that could be construed as a potential conflict of interest.

## Publisher's Note

All claims expressed in this article are solely those of the authors and do not necessarily represent those of their affiliated organizations, or those of the publisher, the editors and the reviewers. Any product that may be evaluated in this article, or claim that may be made by its manufacturer, is not guaranteed or endorsed by the publisher.
